# Liver Abscess Formation After Laparoscopic Radiofrequency Ablation of Metastatic Colon Cancer

**DOI:** 10.7759/cureus.27556

**Published:** 2022-08-01

**Authors:** Arthur Cecchini, Ahmad Othman, Ryan Burgess, Madeeha S Sadiq, Amanda Cecchini

**Affiliations:** 1 Internal Medicine, East Tennessee State University Quillen College of Medicine, Johnson City, USA; 2 Pulmonary and Critical Care Medicine, Eastern Virginia Medical School, Norfolk, USA

**Keywords:** thermal radiofrequency ablation, metastatic liver lesion, invasive colon cancer, colon cancer, liver abscess drainage, liver abscess

## Abstract

Radiofrequency ablation (RFA) may be used to treat either benign or malignant tumors of the liver. Complications are relatively rare, with the most common being pyogenic liver abscess formation. Risk factors for pyogenic liver abscess formation include Child-Pugh class B or C cirrhosis, biliary tract disease, diabetes mellitus, and preexisting biliary diversion. Differentiating sterile post-ablative necrosis from abscess formation may be difficult on imaging as air may be considered a normal post-ablation finding. Treatment of pyogenic liver abscesses includes drainage and antibiotics targeting the most common organisms. This case presents a 71-year-old female with none of the above risk factors who developed a pyogenic liver abscess after undergoing RFA for a solitary liver metastasis secondary to biopsy-proven colonic adenocarcinoma. She was successfully treated with antibiotics and an indwelling percutaneous drain.

## Introduction

Tumor ablation techniques, such as radiofrequency ablation (RFA) and microwave ablation (MWA), are often used as first-line treatments for various types of primary liver tumors or metastatic tumors to the liver [1]. Patients who are not candidates for surgical intervention of hepatic lesions that would typically be treated by resection may also benefit from ablation [2-4].

Multiple studies have shown that thermal ablation of hepatic lesions is generally well-tolerated, and complications are relatively rare [3-6]. When complications arise, liver abscess formation is one of the most common [5,6]. Risk factors for developing a liver abscess after RFA include previous bilioenteric anastomosis, Child-Pugh class B and class C cirrhosis, biliary tract disease, diabetes mellitus, and porta hepatis tumors [7,8].

Liver abscess formation usually presents with nonspecific signs and symptoms, such as right upper quadrant discomfort, fever, elevations in C-reactive protein (CRP), and leukocytosis. Imaging, preferably with computed tomography (CT), is usually required for diagnosis [9,10]. Differentiating abscess formation from sterile necrosis may be difficult on imaging studies as intralesional air may be a normal finding after ablation [11-13]. Treatment is usually with antimicrobial therapy targeted toward enteric organisms and percutaneous drainage [9,10,14].

## Case presentation

**Figure 1:**
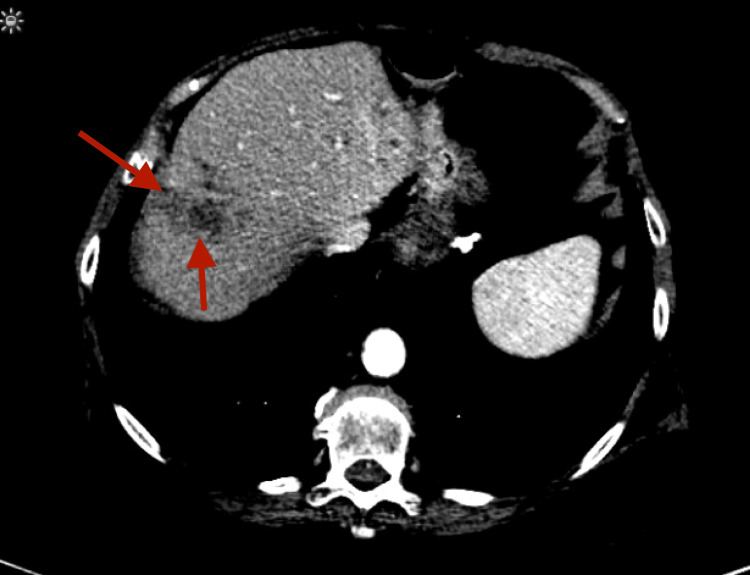
CT of the abdomen and pelvis with the portal venous phase of contrast enhancement showing metastatic adenocarcinoma in liver segment VII (axial view). The study was performed one week before the second RFA. CT: computed tomography; RFA: radiofrequency ablation

A 71-year-old female presented to the hospital with right upper quadrant abdominal pain, weight loss, night sweats, and chills. She was diagnosed with stage IV adenocarcinoma of the ascending colon 20 months ago and subsequently treated with oxaliplatin, 5-fluorouracil, folinic acid, and bevacizumab. Six months later, she underwent a partial colon resection with a primary end-to-end anastomosis, a liver biopsy that showed metastatic adenocarcinoma, and laparoscopic RFA of solitary liver metastasis. Her oxaliplatin was subsequently discontinued because of myelosuppression. A year later, she was found to have a liver mass on imaging for which a computed tomography (CT)-guided biopsy was performed, which showed recurrent metastatic adenocarcinoma (Figure 1).

**Figure 2:**
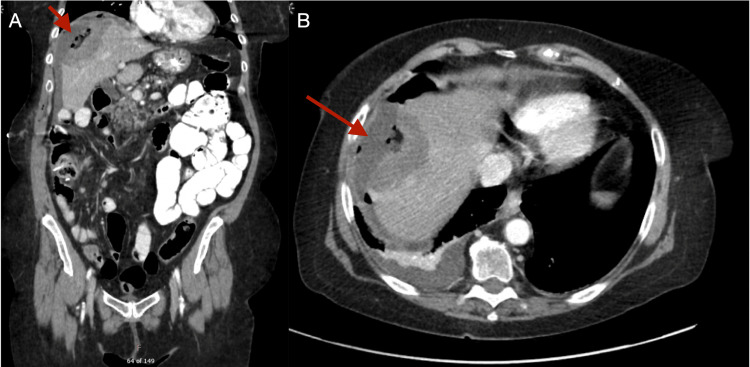
CT of the abdomen and pelvis with the portal venous phase of contrast and oral contrast enhancement performed three days post RFA showing a 6.2 × 4.1 × 7 cm area of low attenuation with associated fluid and pneumatosis. (A) Coronal view. (B) Axial view. CT: computed tomography; RFA: radiofrequency ablation

Another laparoscopic RFA of the lesion was performed along with imaging three days post-procedure which showed expected post-ablation changes (Figures 2A, 2B).

**Figure 3:**
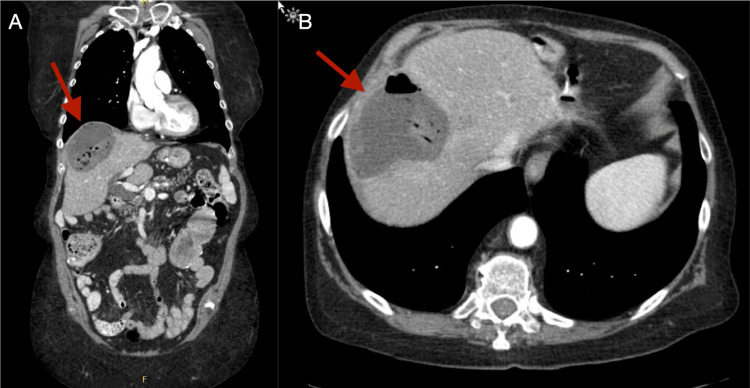
CT of the abdomen and pelvis with the portal venous phase of contrast enhancement performed two months after RFA (one week before hospital admission) showing a persistent necrotic mass with gas densities. (A) Coronal view. (B) Axial view. CT: computed tomography; RFA: radiofrequency ablation

She was seen two months later by her outpatient oncologist for worsening right upper quadrant abdominal pain and night sweats. She was afebrile and her complete blood count (CBC) and complete metabolic panel (CMP) were unremarkable. A C-reactive protein (CRP) level was not obtained. A CT scan of the abdomen revealed coagulative necrosis with gas densities where the metastasis was previously located (Figures 3A, 3B). The changes were determined to be consistent with her previous ablation.

Her home opioid dose was escalated to control her discomfort. A week later, she presented to our hospital with progressive right upper quadrant pain, weight loss, chills, and night sweats.

On admission, vital signs showed blood pressure of 184/92 mmHg, a heart rate of 80 beats per minute, a temperature of 97.6°F, respiratory rate of 18 breaths per minute, and oxygen saturation of 98% on room air. Physical examination revealed right upper quadrant tenderness, a healed surgical scar, and no rebound tenderness. Her port site was intact without erythema or induration. The rest of the examination was unremarkable. Laboratory studies on admission are shown in Table 1.

**Table 1:** Laboratory studies on admission.

Laboratory studies	Patient values	Reference values
Sodium (mmol/L)	128	136–145
Potassium (mmol/L)	4.0	3.5–5.0
Chloride (mmol/L)	98	98–106
Bicarbonate (mmol/L)	21	22–32
Glucose (mg/dL)	69	70–99
Blood urea nitrogen (mg/dL)	19	6–20
Creatinine (mg/dL)	1.03	0.60–1.10
Calcium (mg/dL)	8.0	8.8–10.2
Anion gap (mmol/L)	9	5–15
Total protein (g/dL)	6.4	6.0–8.0
Albumin (g/dL)	3.1	3.5–5.2
Alanine aminotransferase (U/L)	20	14–54
Aspartate aminotransferase (U/L)	26	15–41
Alkaline phosphatase (U/L)	187	39–117
Total bilirubin (g/dL)	0.9	0.3–1.0
Lactic acid (mmol/L)	1.0	0.7–2.1
Magnesium (mg/dL)	1.8	1.5–2.5
Serum osmolality (mOsm/kg H_2_O)	258	266–293
Prothrombin time (seconds)	14.6	9.4–12.5
International normalized ratio	1.3	-
White blood cell count (K/µL)	6.9	4.0–11.0
Red blood cell count (M/µL)	3.99	3.79–5.11
Hemoglobin (g/dL)	12.3	11.7–15.0
Hematocrit (%)	36.3	35–46
Platelet count (K/µL)	166	150–400
Cortisol level, AM (µg/dL)	15	6.7–22.6
Thyroid-stimulating hormone (uIU/ml)	4.66	0.45–5.33
Urine sodium (mmol/L)	87	-
Urine osmolality (mOsm/kg)	684	-

**Figure 4:**
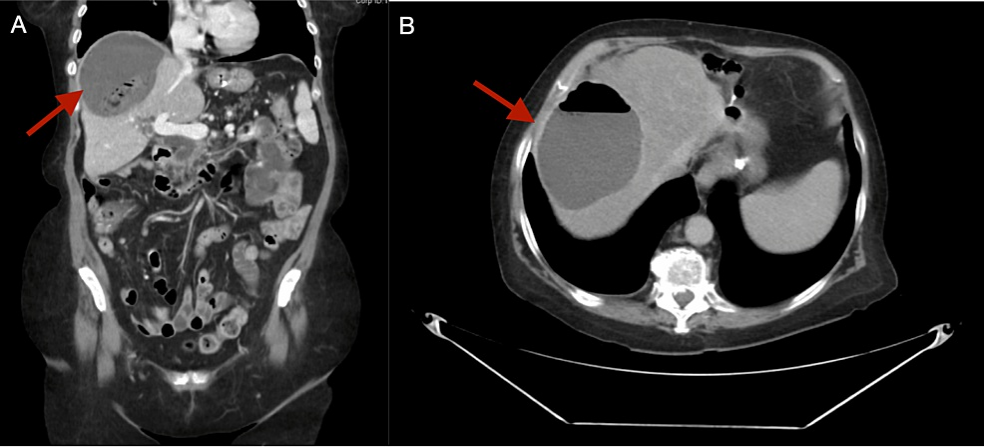
CT of the abdomen and pelvis with the portal venous phase of contrast enhancement performed on admission (one week after the previous CT scan) showing a 10.3 × 8.4 × 9.1 cm air/fluid collection of the liver. (A) Coronal view. (B) Axial view. CT: computed tomography

A new CT scan of the abdomen and pelvis was performed showing an air/fluid collection in the right hepatic lobe (Figures 4A, 4B).

**Figure 5:**
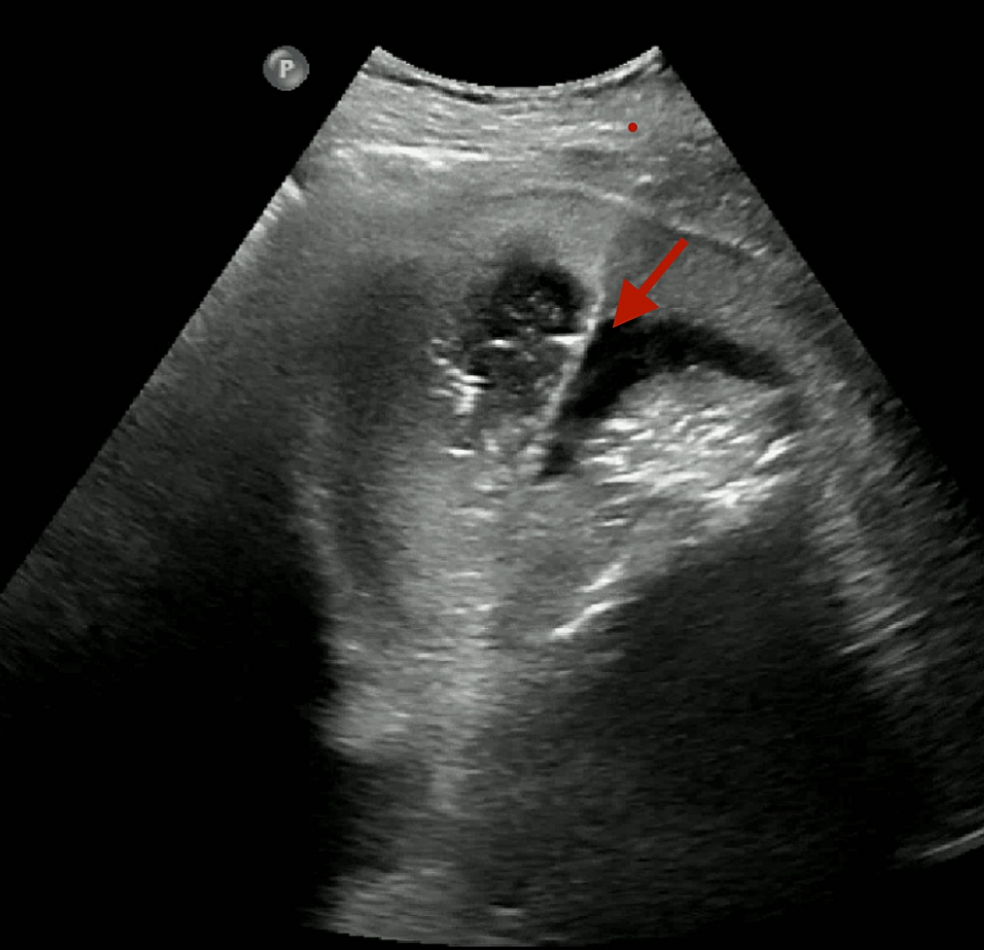
Ultrasound-guided percutaneous drainage of the air/fluid collection (performed within 24 hours of admission).

Two sets of blood cultures were collected followed by piperacillin-tazobactam administration. An interventional radiology consultation was requested for ultrasound-guided drain placement (Figure 5).

**Figure 6:**
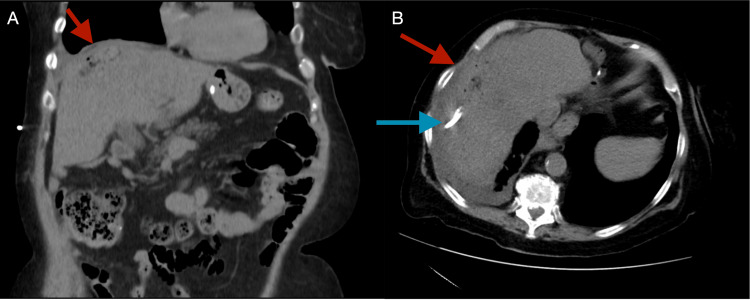
CT of the abdomen without contrast enhancement performed three weeks after drain insertion showing near resolution of the hepatic fluid collection (red arrows) with the drain (blue arrow) before it was removed the same day. (A) Coronal view. (B) Axial view. CT: computed tomography

The catheter was attached to Jackson-Pratt (JP) surgical drain. Aerobic and anaerobic cultures were obtained from the mucopurulent drainage 12 hours after initial antibiotic administration. Both aerobic and anaerobic samples revealed extended-spectrum beta-lactamase-producing (ESBL) *Escherichia coli*. The piperacillin-tazobactam was changed to meropenem, which was switched at discharge to ertapenem for once-daily dosing. The drain was subsequently removed during an outpatient follow-up visit because the drainage had ceased for over a week, her symptoms had resolved, and follow-up imaging revealed a significant decrease in the size of the fluid collection (Figures 6A, 6B).

She ultimately completed a four-week course of parenteral antibiotic therapy and was able to resume her chemotherapy regimen.

## Discussion

Tumor ablation is a minimally invasive technique used to treat tumors in the liver, kidney, lung, or bone. RFA and MWA are two common techniques used to deliver energy to heat the targeted tissue and induce necrosis [1]. Ablation techniques are often used as first-line treatments in patients with benign tumors of the liver, hepatocellular carcinoma (HCC), or metastatic colon cancer of the liver [2,3]. These techniques are also used in patients who are not candidates for surgical resection of liver lesions or as bridge therapy for patients with HCC awaiting liver transplant [2,4]. There are various approaches used for ablation, the most common being laparoscopic, percutaneous, or endoscopic [1].

Despite RFA being usually well-tolerated and considered a relatively low-risk procedure in the treatment of focal liver tumors, there are rare complications that can develop such as portal vein thrombosis, colonic perforation, pleural effusion, pneumothorax, acute renal failure, or liver abscess formation [3,5,6]. Liver abscess formation after RFA is one of the more common complications but still occurs infrequently. In one large single-center study of 1,500 patients, liver abscess formation occurred in 0.6% of patients who underwent RFA [5]. Another study reported liver abscesses occurring in seven of 312 patients, with three of the seven having a previous bilioenteric anastomosis [6]. A third study reported post-procedural liver abscess formation after RFA to be 1.7%, with it being seen more often in patients with Child-Pugh class B or class C cirrhosis, biliary tract disease, diabetes mellitus, or porta hepatis tumors [7]. In patients with preexisting biliary diversion, the risk of developing a liver abscess after RFA is significant and can be 40-50% [8].

The pathogenesis by which liver abscesses develop after RFA is poorly understood, but it is proposed that the most common routes of infection include the hepatic artery, portal vein, and bile ducts [7]. Disrupted biliary epithelium in patients with preexisting bilioenteric anastomosis may attribute to the much higher rates of abscess formation in this subset of patients [6].

Symptoms of liver abscess formation include right upper quadrant abdominal pain, fever, nausea, and vomiting [9,10]. The diagnosis of liver abscess after RFA may be difficult as fever may occur due to the ablation itself. Patients with fever lasting longer than two weeks or a temperature of 38.5°C or greater for more than three days should be screened for the presence of a liver abscess [7].

Signs are nonspecific and include abdominal tenderness and occasionally jaundice. Elevations in CRP, white blood cell counts, and alkaline phosphatase may be present and may help differentiate sterile necrosis from abscess formation [9-11]. Imaging with abdominal US or CT of the abdomen is required for diagnosis, with CT being slightly more sensitive than US (93%-97% versus 82%-95%) [10]. Air bubbles may be present on cross-sectional imaging several days after thermal ablation in patients without abscess formation, thus making the differentiation between abscess and necrotic collection difficult [11-13]. Typically, air should not be visible on cross-sectional imaging beyond one-month post-procedure, and its presence should raise suspicion of abscess formation [12,13].

Many pyogenic liver abscesses are polymicrobial, with the most common organisms isolated being *Escherichia coli*, *Klebsiella pneumoniae*, *Enterococcus*, and *Streptococcus*. *Staphylococcus aureus* is often isolated after cases of penetrating trauma or hepatic chemoembolization [9,10]. *Pseudomonas*, *Citrobacter*, and *Proteus *are less common but have also been isolated [9].

Treatment includes antibiotic therapy against the above organisms and targeted therapy once the culture and sensitivity results are available [9,10]. Abscesses >5 cm or persistent fever are indications for drainage [10]. Drainage catheters are often used for larger abscesses, have a high success rate, and are typically preferred over needle aspiration [14]. Antibiotics are typically continued parenterally for two to three weeks and followed by oral therapy for two to six weeks [10].

## Conclusions

Pyogenic liver abscess formation is the most common, yet relatively infrequent complication after RFA of liver masses. Most patients found to have a liver abscess after RFA will have one or more risk factors, including Child-Pugh class B or C cirrhosis, biliary tract disease, diabetes mellitus, or preexisting biliary diversion. Differentiating post-ablative changes from abscess formation on cross-sectional imaging is difficult, and further studies addressing this would be beneficial. CRP levels may be beneficial in differentiating benign post-ablation changes from infectious processes, especially in patients without fever or leukocytosis. This case shows that clinical suspicion of liver abscess formation after RFA should be high when air is visible at the site of ablation on cross-sectional imaging beyond one month post-procedure.
